# *In silico* region of difference (RD) analysis of *Mycobacterium tuberculosis* complex from sequence reads using RD-Analyzer

**DOI:** 10.1186/s12864-016-3213-1

**Published:** 2016-11-02

**Authors:** Kiatichai Faksri, Eryu Xia, Jun Hao Tan, Yik-Ying Teo, Rick Twee-Hee Ong

**Affiliations:** 1 Department of Microbiology Faculty of Medicine, Khon Kaen University, Khon Kaen, Thailand; 2 Research and Diagnostic Center for Emerging Infectious Diseases (RCEID), Khon Kaen University, Khon Kaen, Thailand; 3 NUS Graduate School for Integrative Sciences and Engineering, National University of Singapore, Singapore, Singapore; 4 Saw Swee Hock School of Public Health, National University of Singapore, Tahir Foundation Building, 12 Science Drive 2, #10-01, Singapore, 117549 Singapore; 5 Department of Statistics and Applied Probability, National University of Singapore, Singapore, Singapore; 6 Life Sciences Institute, National University of Singapore, Singapore, Singapore; 7 Genome Institute of Singapore, Singapore, Singapore

**Keywords:** Region of difference analysis, *Mycobacterium tuberculosis* complex, Whole-genome sequence analysis

## Abstract

**Background:**

Whole-genome sequencing is increasingly used in clinical diagnosis of tuberculosis and study of *Mycobacterium tuberculosis* complex (MTC). MTC consists of several genetically homogenous mycobacteria species which can cause tuberculosis in humans and animals. Regions of difference (RDs) are commonly regarded as gold standard genetic markers for MTC classification.

**Results:**

We develop RD-Analyzer, a tool that can accurately infer the species and lineage of MTC isolates from sequence reads based on the presence and absence of a set of 31 RDs. Applied on a publicly available diverse set of 377 sequenced MTC isolates from known major species and lineages, RD-Analyzer achieved an accuracy of 98.14 % (370/377) in species prediction and a concordance of 98.47 % (257/261) in *Mycobacterium tuberculosis* lineage prediction compared to predictions based on single nucleotide polymorphism markers. By comparing respective sequencing read depths on each genomic position between isolates of different sublineages, we were able to identify the known RD markers in different sublineages of Lineage 4 and provide support for six potential delineating markers having high sensitivities and specificities for sublineage prediction. An extended version of RD-Analyzer was thus developed to allow user-defined RDs for lineage prediction.

**Conclusions:**

RD-Analyzer is a useful and accurate tool for species, lineage and sublineage prediction using known RDs of MTC from sequence reads and is extendable to accepting user-defined RDs for analysis. RD-Analyzer is written in Python and is freely available at https://github.com/xiaeryu/RD-Analyzer.

**Electronic supplementary material:**

The online version of this article (doi:10.1186/s12864-016-3213-1) contains supplementary material, which is available to authorized users.

## Background

Tuberculosis (TB) is a major infectious disease of global public health concern, which resulted in an estimated 9.6 million new cases and 1.5 million deaths worldwide in 2014 [1]. *Mycobacterium tuberculosis* complex (MTC) is the causal agent of TB, which comprises of several genetically homogenous mycobacteria species including human-adapted pathogens of *Mycobacterium tuberculosis* (*Mtb*), *M. africanum*, *M. canettii*, *M. bovis* and animal-adapted pathogens of *M. caprae*, *M. microti* and *M. pinnipedii* which have been reported to cause human infections as well [2]. Recent molecular studies have provided significant insights in revealing the population structure of MTC, identifying distinct MTC lineages associated with geographical regions [3], and have demonstrated that strain diversity is also associated with differences in disease transmissibility, virulence, drug resistance and immune responses [4].

The earliest methods for discriminating strains of clinical MTC isolates relied upon differences in phenotypic characteristics such as colony morphology, which were time consuming, had low discriminatory power, and were not easily performed routinely. Various molecular genotyping techniques for DNA fingerprinting have since been introduced for MTC isolates, including IS*6110*-restriction fragment length polymorphisms (IS*6110-*RFLP) [5], spoligotyping [6] and Mycobacterial interspersed repetitive units-variable number tandem repeats (MIRU-VNTR) [7]. It has been suggested that one of the best strategies for MTC genotyping is using MIRU-VNTR combined with spoligotyping, which can differentiate between clinical isolates to identify disease transmission and outbreak, distinguish between disease relapse and re-infection, and identify contamination [8, 9]. Several studies have however shown that the rapidly evolving genetic markers used in MIRU-VNTR and spoligotyping, although highly discriminatory, are prone to homoplasy or convergent evolution, where the same genetic profile could be obtained in distinct MTC strains that are phylogenetically unrelated, thus confounding strain classification and phylogenetic inference [10–12].

Genotyping methods based on large sequence polymorphisms (LSPs) [13, 14] and single nucleotide polymorphisms (SNPs) [15] are useful tools for phylogenetic study and strain classification of MTC. MTC strains harbor different genomic insertions or deletions called LSPs, which are also known as regions of difference (RDs). In a study of clinical *Mtb* isolates, RDs were demonstrated to be unique event polymorphisms, where the mutations have occurred once in the phylogeny of a species and are thus unique, irreversible, and do not display homoplasy [16]. This is supported by the clonal population structure and lack of horizontal gene transfer events in MTC. RDs can thus be used as robust phylogenetic markers as the deletion of a long genetic sequence in a strain would be inherited and harbored by all descendants of the strain. Specific RDs have been reliably identified in multiple distinct MTC strains, and have been used as gold standard genetic markers for MTC species and phylogenetic lineage prediction [3, 17–19].

Advancements in next-generation sequencing technologies are now enabling whole-genome sequencing (WGS) of MTC clinical isolates to be considered for routine use in the clinical diagnosis of TB, where a single WGS workflow can potentially replace multiple tests currently performed for species identification, phylogenetic strain classification, drug resistance determination and public health molecular epidemiology investigation for disease outbreak and transmission [20, 21]. As a result, bioinformatics tools are needed to translate genomic data into genotypes, especially those obtainable from conventional genotyping techniques to correlate sequenced isolates with previously genotyped MTC isolates that were not sequenced. For genotyping of MTC, *in silico* spoligotyping has been made possible by the development of bioinformatics tools like SpoTyping [22] and SpolPred [23], which serves as the bridge between WGS data and laboratory tests. Other genotyping methods like IS*6110*-RFLP and MIRU-VNTR are difficult to be determined using the short sequence reads generated by the current most widely used sequencing platforms. For phylogenetic study of MTC, SNP-based phylogeny from sequence reads is widely used and various pipelines have been developed for variant calling. However, there is currently no method available for RD analysis from WGS data. Compared to SNP-based lineage prediction, RD has the merits of: (i) being able to classify different MTC species; and (ii) can be easily obtained from both WGS data and current laboratory tests for comparison and validation.

Here we describe RD-Analyzer, a useful tool that accurately infers the species and lineage of MTC isolates from sequence reads based on the presence and absence of a set of 31 RDs. Candidate RDs for more accurate sublineage prediction in Lineage 4 were identified by comparing isolates of different sublineages, which showed high concordance with existing RD markers and provided support for new markers. An extended version of RD-Analyzer was thus developed to accept such user-defined RDs for lineage prediction. The six potential markers identified all showed great sensitivity and specificity in sublineage prediction. RD-Analyzer is written in Python and is freely available [24].

## Implementation

### Informative RD markers for MTC species and *Mtb* lineage/sublineage

Thirty-one RDs were identified in previous studies [3, 17–19] as robust phylogenetic markers that were able to distinguish between distinct species and lineages of MTC. Of these, 13 RDs are relevant for MTC species differentiation, while the remaining 18 are used to resolve different lineages of *Mtb* (Additional file 1: Table S1).

### Longest unique sequence determination for each RD

Genomic sequences of each RD were retrieved from the corresponding genomic regions of the H37Rv genome [GenBank:NC_00962.3]. The RD sequences could span several kilo base pairs, where some fragments may not be unique and have homologous sequences in other regions of the genome. We thus sought to increase the specificity of reads mapping to the RDs by identifying the longest unique sequence (LUS), which is defined as the longest continuous genomic sequence of the RD that is uniquely identified in this RD in all known complete reference genome sequences of MTC species (i.e. *Mtb* H37Rv, *M. canetii* [GenBank:NC019952.1], *M. bovis* [GenBank:BX248333.1], *M. bovis* BCG AF2122/97 [GenBank:NC_002945.3], *M. bovis* BCG *Moreau* [GenBank:AM412059.2], *M. bovis* BCG Pasteur [GenBank:NC_008769.], and *M. africanum* type II [GenBank:NC_015758.1]) and sequence reads of the remaining species where complete genome is not available (i.e., *M. africanum* type I [ENA:ERR400492], *M. microti* [ENA:ERR027295], *M. pinnipedii* [SRA:SRR1239336], and *M. caprae* [SRA:SRR650219]). The catalog of LUS corresponding to each RD used in RD-Analyzer is presented in Additional file 1: Table S1.

### Description of algorithm

**Fig. 1:**
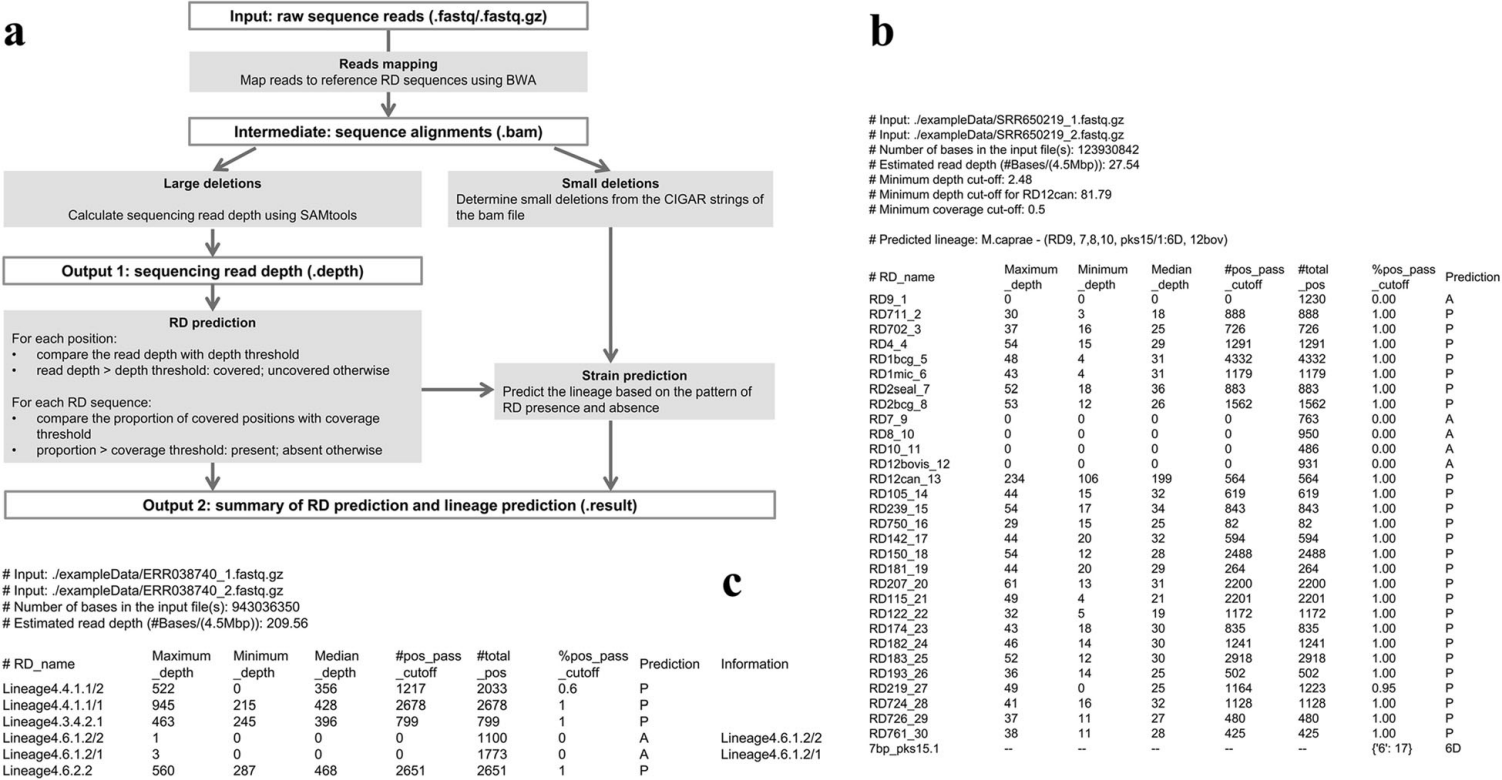
The work flow and output of RD-Analyzer. **a** A schematic representation of the processes in RD-Analyzer. RD-Analyzer accepts sequence reads in FASTQ format. The input sequence reads are mapped to reference RD sequences, after which read depths along the reference sequences would be calculated. Normally, an RD is identified as ‘present’ in the MTC isolate if the median ratio of read depth along the reference sequence is above a specified threshold (default of 0.09 for all RDs, except 2.97 for RD12^can^). The default setting of using the median ratio (ratio at the 50^th^ percentile) can be changed to other percentiles subject to user’s preference. Small RDs are detected from CIGAR strings of mapped reads spanning the potential deletion region. The default RD-Analyzer uses the LUSs of 31 RDs and performs lineage prediction using rules elaborated in Additional file 2: Table S2. The extended version of RD-Analyzer allows for user-defined reference RD sequences without strain prediction. **b** An example output file of default RD-Analyzer. **c** An example output file of extended RD-Analyzer

RD-Analyzer is written in Python and can be used to accurately determine the presence or absence of 31 informative RD markers from raw sequence reads in order to infer the species and lineages of MTC isolates. A schematic representation of the processes in RD-Analyzer is shown in Fig. 1a. RD-Analyzer accepts input files of both single-end and pair-end sequence reads in uncompressed or compressed (using gzip) FASTQ format. The input sequence reads would first be mapped to the LUSs corresponding to each RD using BWA-MEM [25]. Read depths along each LUS would then be calculated using SAMtools [26], which would then be divided by the average sequencing read depth of the isolate estimated from the input sequence reads (number of input sequence bases divided by 4.5 million base pairs) to be transformed into a ratio to eliminate the effect of sequencing throughput. Normally, an RD is identified as ‘present’ in the MTC isolate if the median ratio of read depth along the LUS is above a specified threshold (default value of 0.09 for all RDs, except for RD12^can^ where 2.97 is used). The default setting of using the median ratio (ratio at the 50^th^ percentile) can be changed to other percentiles subject to user’s preference. RD *pks15/1* can be present in three possible genotypes: (i) complete form; (ii) incomplete form with a 6 base pair deletion, or (iii) incomplete form with a 7 base pair deletion. To determine the genotype of RD *pks15/*1, CIGAR strings of mapped reads spanning the potential deletion region would be examined in the BAM file, where deletions at base positions from 152 to 167 of the LUS sequence would be translated to the corresponding genotype. The presence or absence of each RD would be summarized in the output file with the extension of ‘.result’ (Fig. 1b). The lineage of the isolate would then be predicted based on the deletion of specific RDs as elaborated in Additional file 2: Table S2.

The extended version of RD-Analyzer is designed to accept user-specified RD sequences instead of using the LUSs identified for the 31 known RDs with similar processing steps except that only RD prediction for presence and absence but not strain prediction would be performed. An example output of the extended RD-Analyzer is shown in Fig. 1c.

### Description of dataset

Analysis and assessment using RD-Analyzer in this study were based on 377 diverse sequenced MTC clinical isolates from known major species and lineages in public databases [27–30]. The MTC isolates span the major MTC species (261 *Mtb,* 30 *M. africanum*, 17 *M. canettii,* 54 *M. bovis*, 4 *M. caprae*, 5 *M. microti*, and 6 *M. pinnipedii*) and the main *Mtb* lineages (31 Lineage 1, 48 Lineage 2, 65 Lineage 3, and 117 Lineage 4). They were sequenced with varying characteristics of: (i) sequencing platforms of Illumina HiSeq, MiSeq and Genome Analyzer; (ii) either single-end reads and paired-end reads; and (iii) a wide range of sequencing read depths ranging from 5X to 600X. The sequence reads of these isolates were downloaded from European Nucleotide Archive (ENA) and NCBI Sequence Read Archive (SRA), of which the details are presented in Additional file 3: Table S3. No sequence reads for *M. bovis* BCG strains were available. We thus simulated the sequence reads using the ART read simulator [31]. Three complete *M. bovis* BCG genomes were each used as a reference sequence to generate sequence reads: (i) *M. bovis* AF2122/97; (ii) *M. bovis* BCG str. Moreau RDJ; and (iii) *M. bovis* BCG Pasteur 1173P2. For each reference sequence, pair-end reads of 250 and 100 bp were each generated at read depths of 50X and 100X.

The validation dataset included 100 randomly selected MTC isolates that cover major *Mtb* lineages and sublineages based on SNP markers, with publicly available sequence reads (Additional file 4: Table S4).

### Threshold selection

The default threshold to determine an RD as present or absent in a sequenced isolate was selected based on the 377 diverse MTC isolates mentioned above. Two variables were used for each RD in each isolate to assess the optimal threshold: (i) the ratio between the median read depth of the corresponding LUS and the estimated sequencing read depth of the isolate, which was used as the explanatory variable for classification; and (ii) the fact of the presence or absence of the RD, which was used as the outcome of classification. The fact of the presence or absence of RDs distinguishing MTC species was defined based on phenotypic assays and molecular technique from previous studies [27–30], while the fact of the presence or absence of RDs for *Mtb* lineage and sublineage prediction was defined based on the results of TB-profiler [32] that makes use of a robust SNP marker set for *Mtb* classification. A set of different ratios was used as the thresholds for RD prediction to assess the performance of the classifier. A receiver operating characteristic (ROC) curve was plotted to show the true positive rate (TPR) against the false positive rate (FPR) at various threshold settings. AUC was calculated as the area under the ROC curve. The default threshold was selected to weigh both TPR and FPR equally, thus being the threshold that could minimize the value of FPR^2^ + (1-TPR)^2^.

### Performance assessment

The performance of RD-Analyzer in species, lineage and sublineage prediction was assessed based on the dataset of 377 diverse MTC species and *Mtb* strains mentioned above. The performance of RD-Analyzer in RD prediction was assessed based on an independent validation dataset described above. In all assessments, RD-Analyzer was run using default settings.

### Detection of potential RDs for sublineage classification in Lineage 4 *Mtb* isolates

Of the 377 MTC clinical isolates, 117 were predicted to be of Lineage 4 based on the robust SNP marker set [15] and were used in the detection of potential RDs for sublineage classification. Thirteen sublineages in Lineage 4 had more than 4 isolates in the studied dataset, of which six sublineages have well defined RD markers for classification (Lineage 4.8, 4.1.2.1, 4.3.3, 4.1.1.3, 4.1.1.1, and 4.5), while seven sublineages do not have such an RD marker (Lineage 4.3.4.2.1, Lineage 4.6.1.2, Lineage 4.3.4.1, Lineage 4.6, Lineage 4.6.2.2, Lineage 4.4.1.1, and Lineage 4.2.2).

For the 117 isolates, sequence reads of each isolate were aligned to the H37Rv reference sequence to calculate the read depth for each position, which was later divided by the average sequencing read depth of the isolate to be transformed into a ratio. A comparison was conducted for each sublineage, where isolates of this sublineage were used as the experiment group while isolates of other sublineages were used as the control group. In the comparison, the mean depth (represented as a ratio) of the experiment group would be compared with the mean depth of the control group using *t*-test position by position, producing *p*-values indicating the significance in the difference of the average depth between the two groups. Consecutive regions with low *p*-values are thus possible candidate RD markers for sublineage classification. In our analysis, consecutive positions having -log_10_ (*p*-value) larger than 60 were taken as a candidate marker, resulting in candidate RD markers for 10 of the 13 sublineages.

For sublineages with robust RD markers, the identified RDs were compared with existing markers to assess the validity of this identification method. For sublineages without robust RD markers, the longest identified RDs were used as the reference RD sequences to be searched in the 117 isolates using extended RD-Analyzer, where the classification sensitivity and specificity of each RD marker would be calculated based on the output.

## Results

### Threshold selection in RD-Analyzer

**Fig. 2:**
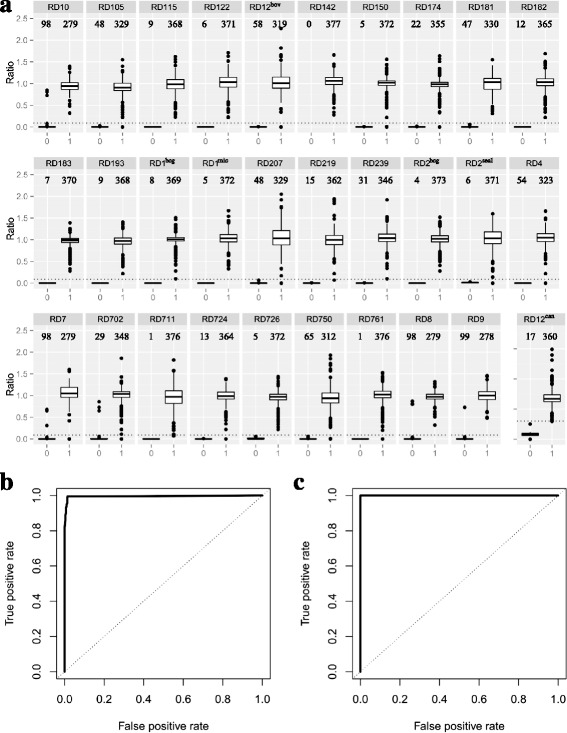
Threshold selection in RD-Analyzer. **a** Different ratios of read depths between present and absent RDs. The ratio refers to the ratio between the median read depth along the RD sequence and estimated genome read depths. The *dotted lines* indicate the optimal threshold of the read depth (0.09 for all RDs, except 2.97 for RD12^can^). The numbers above the boxes indicate the number of instances included in the *box*. **b** ROC curve for threshold selection for RDs except RD12can. The ROC curve shows very high TPR and very low FPR at nearly all thresholds with the area under the curve being 0.9907. The *dotted diagonal line* is the line of no discrimination. The default threshold was selected to be 0.09, which produced a TPR of 0.9949 and an FPR of 0.9856. **c** ROC curve for threshold selection for RD12^can^. The ROC curve has an AUC of 1. The default threshold was selected to be 2.97, producing a TPR of 1.0000 and an FPR of 1.0000

Determining an RD as ‘present’ or ‘absent’ is generally a binary classification problem on a univariate dataset. The explanatory variable for classification is the median ratio, where a ratio is calculated as read depth on a specific position of the corresponding LUS divided by the estimated average sequencing read depth of the isolate. The outcome for classification is the fact of the presence or absence of the RD. In general, the ratios of two outcomes (‘Presence’ or ‘Absence’) differed greatly. In Fig. 2a, the distribution of ratios grouped by the outcome for each RD was summarized as boxplots, where clear differences were observed. RD12^can^ was plotted separately because the ratios have a different scale compared to other RDs, and was thus optimized separately with a different threshold. Both ROC curves (Fig. 2b for all RDs excluding RD12^can^, and Fig. 2c for RD12^can^) show very high TPR and very low FPR at nearly all thresholds with the AUC being 0.9907 and 1, respectively. The default thresholds were selected to be 0.09 for all RDs, which produced a TPR of 0.9949 and an FPR of 0.9856, except for RD12^can^, where 2.97 was used and a TPR of 1.0000 and an FPR of 1.0000 were produced.

### Performance assessment of RD, species, lineage, and sublineage prediction

RD-Analyzer was run on the dataset of 377 isolates using default settings to assess the accuracy in predicting species, lineages and sublineages (Table 1). RD-Analyzer achieved an accuracy of 98.14 % (370/377) in species prediction. Of the seven discordant species predictions, two are Lineage 3 *Mtb* isolates that have the typical *M. bovis* deletion RD4, three are *M. africanum* isolates that do not have the typical *M. africanum* deletions of RD7, RD8, RD9, and RD702, one *M. canettii* isolate has the typical *Mtb* Lineage 3 deletion RD105, and one *M. caprae* isolate was sequenced at extremely low sequencing depth. For *Mtb* lineage/sublineage prediction, a concordance of 98.47 % (257/261) was reported. Homoplasy was observed in 4 isolates, resulting in reports of more than one lineage (Additional file 3: Table S3).

**Table 1:**
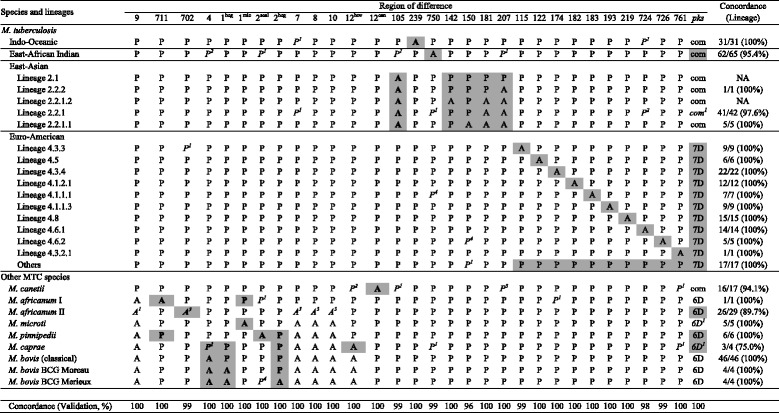
Performance of RD-Analyzer for predicting RD and differentiating *Mtb* lineages and MTC species

Note *A* absence, *P* presence, *com* complete (no deletion), *7D* 7 bp deletion, *6D* 6 bp deletion; and *NA* not available. Concordance (Validation, %): concordance in predicting respective RDs in the validation dataset, in the unit of %. Concordance (Lineage): concordance of lineage/sublineage prediction for each lineage. Bold letters with grey shades refer to key makers for species or lineages identification. Italic characters denote where unexpected absence and presence was discovered, with the superscript denotes the number of strains with unexpected predictions

RD-Analyzer was also run on an independent validation dataset of 100 *Mtb* isolates to assess the performance in predicting RD, and lineage/sublineage. RD-Analyzer achieved an average accuracy of 99.64 % in predicting RDs (ranging from 96 to 100 % for different RDs, Table 1 and Additional file 4: Table S4), and an accuracy of 97 % (97/100) in predicting lineage/sublineage.

### Detection of potential RDs for sublineage classification in Lineage 4 *Mtb* isolates

Though the 31 RD markers used in RD-Analyzer can accurately predict MTC species and *Mtb* lineage, they have lower discriminatory power than SNP markers. Some sublineages with robust SNP marker cannot be distinguished using existing RDs. We thus made the attempt to identify RDs that can potentially be markers for sublineage classification.

**Fig. 3:**
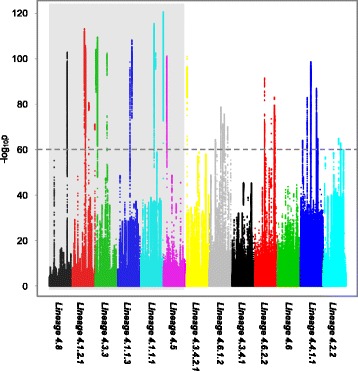
Detection of potential RDs for sublineage classification in Lineage 4 *Mtb* isolates. In the detection of potential RDs for a certain sublineage, isolates belong to this sublineage constitute the experiment group while other isolates constitute the control group. For each sublineage, the *p*-values reflecting the difference in the read depth between the experiment group and the control groups were calculated for each position and translated into –log_10_ (*p*-value) to be plotted on the y-axis of the plot, where the x-axis is grouped by the studied sublineage and the values indicate the genomic positions along the reference genome. Extremely low *p*-values are indicative of significant difference in read depth between the two groups. Regions with consecutive positions having –log_10_ (*p*-value) larger than 60 were regarded as candidate RD markers. Those sublineages with well-defined RD makers are *shaded gray* in the background

Of the 117 Lineage 4 *Mtb* clinical isolates, 13 sublineages had more than 4 isolates in the studied dataset and were thus included in the analysis. Using our criteria, potential RD markers have been identified in 10 of the 13 sublineages (Fig. 3). For sublineages with well-defined RD markers, the RDs identified were compared with existing markers (Table 2). In all the six sublineages with existing markers, the longest RDs identified using our method were nearly identical to existing markers, which is a strong indication of the effectiveness of our detection method. Out of the seven sublineages without well-defined RD markers, four has at least one RD identified as potential RD maker (Table 3). Lineage 4.3.4.2.1, Lineage 4.6.1.2, Lineage 4.6.2.2 and Lineage 4.4.1.1 have one, five, four, five such regions detected, respectively. For sublineages with more than one region identified, regions longer than 1000 bp were taken as potential RD markers.

**Table 2:** Identification of potential RDs for lineage classification in *Mtb* lineages with existing RD markers

Lineage	Sample size	Existing RD markers			RD detected			
		Name	Start	End	No.	Start	End	Length (bp)
Lineage 4.8	15	**RD219**	**3,448,504**	**3,451,396**	**1**	**3,448,497**	**3,451,398**	**2,902**
Lineage 4.1.2.1	12	**RD182**	**2,545,194**	**2,551,674**	**1**	**2,545,195**	**2,551,675**	**6,481**
					2	2,361,910	2,363,682	1,773
					3	3,194,709	3,194,793	85
					4	4,375,626	4,375,708	83
Lineage 4.3.3	9	**RD115**	**453,364**	**455,971**	**1**	**453,367**	**455,972**	**2,606**
					2	171,458	171,778	321
					3	2,306,444	2,306,724	281
Lineage 4.1.1.3	9	**RD193**	**2,704,306**	**2,704,807**	**1**	**2,704,310**	**2,704,806**	**497**
					2	2,339,260	2,339,402	143
Lineage 4.1.1.1	7	**RD183**	**2,585,853**	**2,588,770**	**1**	**2,585,856**	**2,588,768**	**2,913**
					2	4,370,424	4,373,233	2,810
					3	2,866,744	2,866,852	109
Lineage 4.5	6	**RD122**	**669,793**	**670,964**	**1**	**669,795**	**670,965**	**1,171**

Note Bold letters emphasize the concordance between exiting RD markers and RDs detected. The start and end positions correspond to the genomic positions of *Mtb* H37Rv genome

**Table 3:** Identification of potential RDs for lineage classification in *Mtb* lineages without existing RD markers

Lineage	Sample size	RD detected				Performance	
		No.	Start	End	Length (bp)	Sensitivity	Specificity
Lineage 4.3.4.2.1	15	**1**	**164,942**	**165,740**	**799**	**100 % (15/15)**	**100 % (102/102)**
Lineage 4.6.1.2	11	**1**	**2,902,556**	**2,904,328**	**1,773**	**100 % (11/11)**	**97.17 % (103/106)**
		**2**	**2,265,142**	**2,266,241**	**1,100**	**100 % (11/11)**	**98.11 % (104/106)**
		3	1,190,141	1,190,733	593		
		4	2,634,174	2,634,542	369		
		5	3,594,343	3,594,407	65		
Lineage 4.6.2.2	4	**1**	**1,897,554**	**1,900,204**	**2,651**	**100 % (4/4)**	**99.12 % (112/113)**
		2	3,785,220	3,785,638	419		
		3	3,905,337	3,905,721	385		
		4	3,742,614	3,742,895	282		
Lineage 4.4.1.1	4	**1**	**2,025,760**	**2,028,437**	**2,678**	**100 % (4/4)**	**99.12 % (112/113)**
		**2**	**3,123,673**	**3,125,705**	**2,033**	**100 % (4/4)**	**99.12 % (112/113)**
		3	3,112,311	3,112,459	149		
		4	1,313,135	1,313,278	144		
		5	3,377,623	3,377,670	48		

Note Bold letters refer to the potential RD markers whose sensitivity and specificity for classification have been assessed. The start and end positions correspond to the genomic positions of *Mtb* H37Rv genome

An extended version of RD-Analyzer was used to identify the potential RD markers in all the 117 Lineage 4 *Mtb* isolates to assess the sensitivity and specificity using these markers for classification. The results demonstrated that a 799 bp deletion served as a marker for Lineage 4.3.4.2.1 at 100 % sensitivity and specificity. Two markers, each of 1773 and 1100 bp in length, served as markers for Lineage 4.6.2.1 both at sensitivity of 100 % and specificity of 97.17 and 98.11 % respectively. One 2651 bp deletion identifies Lineage 4.6.2.2 at a sensitivity of 100 % and a specificity of 99.12 %. The two markers, 2678 and 2033 bp in length, respectively, both detected Lineage 4.4.1.1 *Mtb* isolates at a sensitivity of 100 % and a specificity of 99.12 %. These are candidate RD markers for *Mtb* sublineage prediction that may be useful in increasing the resolution of MTC classification using RD, though not validated by laboratory tests.

## Discussion

With the advancements in sequencing technology, WGS has become more accessible to microbiology laboratories for clinical diagnosis of TB and study of MTC. Though RD is considered as the gold standard for molecular genotyping of MTC species and *Mtb* lineage determination, no *in silico* tool is available for RD analysis. In this study, we present a new bioinformatics tool, RD-Analyzer, for *in silico* RD analysis and genotyping of MTC clinical isolates from raw sequence reads. When using default settings, RD-Analyzer can accurately infer the species and lineage of MTC based on the presence and absence of a set of 31 RDs well described for MTC classification. A useful method was also described in this study to identify potential RD markers for lineage identification. We thus extended the use of RD-Analyzer to allow user-specified RDs to be used.

Analysis of RDs can provide useful information for WGS analysis of MTC. In the practical workflow for rapid diagnosis of TB based on WGS, the DNA would be extracted from automated liquid culture with positive result of acid fast bacilli staining and submitted for high-throughput sequencing. After obtaining the raw sequence reads, *in silico* spoligotyping tools like SpoTyping and SpolPred can be used to differentiate MTC from nontuberculous mycobacteria (NTM) as the direct repeat region assessed in spoligotyping is specific for MTC and not found in NTM. The next step would be differentiating the species of MTC. Though SNPs are useful for phylogenetic study of *Mtb*, SNP markers presently cannot be used to distinguish all reported MTC species [32, 33]. While spoligotyping patterns can be used to identify species if a specific pattern has previously been reported for a certain species, the determination would be based on searching the database, and some patterns may not have been reported before. RD is therefore a useful marker that can be used to differentiate MTC species, where certain deletions are indicative of specific species. If the clinical isolate is *Mtb*, lineages and sublineages would be identified as a routine. SNPs and RDs are both regarded as gold standard for lineage identification, which are well correlated with each other. The advantages of SNPs are that: (i) the current SNP sets can provide higher resolution than the RD set; and (ii) homoplasy is less observed in robust SNP sets. Though having lower discriminatory power than SNP markers, the RD markers used in RD-Analyzer can accurately predict MTC species and *Mtb* lineage. Here, we have also reported a method for determination of potential RD markers for lineage prediction from sequence reads, we could thus not only discover more informative RDs to increase the classification resolution, but also propose candidate RDs for further laboratory verification on a larger dataset.

Deletion of certain RDs is causing structural variation, which can be determined by detecting structural variants in WGS analysis. However, such determinations are not targeted, need post-processing to determine the absence or presence of the RDs, and are difficult to incorporate into the comprehensive WGS pipeline for MTC. We thus streamline the process to a more targeted approach as a pipeline that not only determines the RD patterns but also performs species and lineage predictions based on the patterns.

RDs are useful markers but are not perfect. We have observed occurrences of homoplasy in our analysis, which could either be due to true homoplasy present in the sample that is sporadic deletion of RDs or as a result of an erroneous identification made by RD-Analyzer. It is possible that RD-Analyzer make a prediction of presence of an RD where the actual biology of the bacilli is absence due to similar genomic sequences in the genome, unspecific reads mapping, and false read depth calculation. Attempts were made to solve this problem by excluding RD sequences that are not unique in reported reference sequences and using the LUSs in the detection. It is less likely that RD-Analyzer will falsely determine an RD to be absent or deleted when it is present provided the sample is sequenced to a sufficient throughput and read depth (>10X read depth, for example).

The technical limitations of RD-Analyzer were also noted. First, RD-Analyzer did not work well with isolates sequenced at low read depth. For example, RD-Analyzer presented unexpected RD patterns when used on an *M. caprae* isolate [SRA:SRR650226] sequenced at ~5X read depth. Second, though we have made efforts to eliminate unspecific reads mapping by identifying LUSs in a sufficiently diverse and moderate number of sequenced MTC isolates, there is no guarantee of the uniqueness of the LUSs in newly sequenced isolates. Third, RD-Analyzer can fail to differentiate between mixed infections as deletion in one strain may be compensated by reads from another strain, thus making an incorrect inference of presence of the RD.

In this study, we have explored the identification of potential RD markers for sublineage prediction using 117 Lineage 4 *Mtb* isolates. Though the sample sizes for each sublineage were not large, results were clear and showed patterns of specific sequence deletions in some sublineages. The effectiveness of this identification method was well demonstrated by the perfect concordance between the potential RD markers determined using this method and the well-defined RD markers in sublineages with defined RD markers. This method thus has high potential to be used for detecting novel RDs for sublineage identification. Assessment of the potential RD markers identified showed the markers are highly sensitive though some are not 100 % specific. Those RDs are also potentially useful to inform laboratory tests to determine lineage and sublineage of *Mtb* isolates without using WGS.

## Conclusions

RDs are robust markers for MTC strain identification and can be analyzed and determined using sequence reads. RD-Analyzer is a useful tool for accurate species, lineage and sublineage prediction using known RDs of MTC from sequence reads and is extendable to user-defined RDs.

## Additional files

Additional file 1: Table S1. Informative RD for MTC analysis and its characteristics. (XLS 25 kb)
Additional file 2: Table S2. Prediction rule for MTC species and *Mtb* lineages/sublineages. (XLS 25 kb)
Additional file 3: Table S3. Raw data and outputs from RD-Analyzer and characteristics of WGS from *Mtb* lineage and sublineages and other species of MTC retrieved from previous studies and public databases. (XLS 224 kb)
Additional file 4: Table S4. Outputs from RD-Analyzer in the validation set of *Mtb* isolates. (XLS 107 kb)

## Abbreviations

FPR: False positive rate
IS*6110*-RFLP: IS6*110*-restriction fragment length polymorphisms
LSP: Large sequence polymorphism
LUS: Longest unique sequence
MIRU-VNTR: Mycobacterial interspersed repetitive units-variable number tandem repeats
*Mtb*: *Mycobacterium tuberculosis*
MTC: *Mycobacterium tuberculosis* complex
NTM: Nontuberculous mycobacteria
RD: Region of difference
ROC: Receiver operating characteristic
SNP: Single nucleotide polymorphism
TB: Tuberculosis
TPR: True positive rate
WGS: Whole-genome sequencing
